# miR-665通过靶向调控LLGL1促进小细胞肺癌生物学行为的研究

**DOI:** 10.3779/j.issn.1009-3419.2020.104.03

**Published:** 2020-04-20

**Authors:** 荣凤 刘, 玲玲 张, 志宏 徐, 彦芝 崔

**Affiliations:** 050011 石家庄，河北医科大学第四医院肿瘤内科 Department of Medical Oncology, The Fourth Hospital of Hebei Medical University, Shijiazhuang 050011, China

**Keywords:** 小细胞肺癌, miR-665, LLGL1, Small cell lung cancer, miR-665, LLGL1

## Abstract

**背景与目的:**

MicroRNAs（miRNAs）是一种广泛存在于真核生物体中的非编码小分子RNA，尽管一些miRNA在肿瘤中作用已被发现，但是miR-665对小细胞肺癌的中的表达及影响还尚不清楚。本研究旨在分析miR-665对肺癌细胞增殖、周期、侵袭和迁移的影响，探讨miR-665在小细胞肺癌中发挥的作用及其工作机制。

**方法:**

qRT-PCR检测miR-665在肺癌组织和癌旁正常组织中的表达水平；TargetScan预测miR-665的潜在靶基因并用双荧光素酶报告基因实验、qRT-PCR和Western blot进行验证；免疫组化、qRT-PCR和Western blot检测LLGL1在肺癌组织和癌旁正常组织中的表达水平；CCK8法、流式细胞法、Transwell和细胞划痕实验检测miR-665和LLGL1对肺癌细胞NCI-H446、NCI-H1688增殖、侵袭、迁移以及S期细胞比值的影响；构建肺癌裸鼠移植瘤模型并观察miR-665对小鼠肿瘤生长的影响。

**结果:**

miR-665在肺癌组织中的表达水平明显高于癌旁正常组织；miR-665能靶向作用于LLGL1的3’-UTR并抑制其表达；相比于癌旁正常组织，LLGL1在肺癌组织中的表达水平明显降低；抑制miR-665的表达可以抑制肺癌NCI-H446细胞的增殖、S期细胞比值、侵袭和迁移能力，而干扰LLGL1能逆转这种抑制效果；上调miR-665则促进肺癌NCI-H1688的增殖、S期细胞比值、侵袭和迁移能力，但这种促进效果同样被LLGL1的过表达逆转；在肺癌裸鼠移植瘤模型中，抑制miR-665能上调LLGL1蛋白的表达并抑制肿瘤的生长，而上调miR-665的表达则可以产生相反的结果。

**结论:**

miR-665表达水平的变化与肺癌密切相关，miR-665可以通过抑制其靶基因*LLGL1*的表达促进肺癌细胞的生物学行为，在小细胞肺癌中发挥促癌基因的作用。

## Abstract (en)

**Background and objective:**

MicroRNAs (miRNAs) are non-coding small molecule RNAs that are widely found in eukaryotic organisms, although some miRNAs have been found in tumors, the expression and effects of miR-665 on small cell lung cancer (SCLC) are unclear. The aim of this study was to analyze the effects of miR-665 on proliferation, cycle, invasion and migration of SCLC cells, and to explore the role of miR-665 in SCLC and its working mechanism.

**Methods:**

The expression of miR-665 in SCLC tissues and adjacent normal tissues was detected by qRT-PCR. TargetScan predicted potential target genes for miR-665 and validated with dual luciferase reporter assays, qRT-PCR and Western blot. CCK8 assay, flow cytometry, Transwell and wound healing assay to detect the effects of miR-665 and LLGL1 on proliferation, invasion, migration and S-phase fraction of SCLC cell line NCI-H446, NCI-H1688. A nude mouse xenograft model of SCLC was constructed and the effect of miR-665 on tumor growth in mice was observed.

**Results:**

The expression of miR-665 in SCLC tissues was significantly higher than that in non-tumor normal tissues. MiR-665 could target 3'-UTR of LLGL1 and inhibit its expression. Compared with non-tumor normal tissues, the expression of LLGL1 was significantly lower in SCLC tissues. Inhibition of miR-665 expression could inhibit proliferation, S-phase fraction, invasion and migration ability of SCLC NCL-H446 cells, and interference LLGL1 expression could reverse this inhibition effect. Up-regulation of miR-665 expression could promoted proliferation, S-phase fraction, invasion and migration ability of SCLC NCI-H1688 cells, but this promotion effect was also reversed by overexpression of LLGL1. In a nude mouse xenograft model of SCLC, inhibition of miR-665 expression could up-regulate LLGL1 protein expression and inhibit tumor growth, while up-regulation of miR-665 expression could produce opposite results.

**Conclusion:**

The expression of miR-665 is closely related to SCLC. miR-665 can promote the biological behavior of SCLC cells by inhibiting the expression of target gene *LLGL1*, and miR-665 play a role in tumor-promoting genes in SCLC.

肺癌是当今世界上对人类健康危害最大的恶性肿瘤之一，小细胞肺癌（small cell lung cancer, SCLC）是一种侵袭性恶性肿瘤，约占肺癌的13%，侵袭力强，恶性程度高^[1]^。其临床特点为：肿瘤细胞倍增时间短，进展快，常伴内分泌异常或类癌综合征^[2]^。幼虫巨大致死基因（Lethal giantlarvae, *Lgl*）是在果蝇里发现的抑癌基因，目前已经发现Lgl在人类的同源基因*LLGL1*也同样担负着抑癌基因的作用^[3]^。人*LLGL1*基因位于17号染色体近着丝点区，近来的分子学研究显示这一区域可能存在与神经外胚层肿瘤相关基因。目前已有报道^[4-6]^显示LLGL1在乳腺癌、胃癌等多种癌症中表达下降或不表达，提示LLGL1在肿瘤发生发展过程中起重要作用。miRNA是一类具有蛋白调控功能的内源性非编码小分子RNA，长度大约22个核苷酸。miRNA参与细胞发育、器官生成、细胞增殖及凋亡等多种细胞生命活动，并且在控制肿瘤的起始和进展中发挥重要作用^[7, 8]^。现己发现不同的miRNA在多种肿瘤疾病中具有不同功能，可能为癌基因，降低其表达能够抑制肿瘤细胞的生长；也可能为抑癌因子，降低其表达会导致肿瘤的发生^[9, 10]^。因此对miRNA深入研究可以为肺癌的诊断及治疗提供新的方向。本研究旨在探究miR-665在SCLC中的作用，分析miR-665通过靶向调控LLGL1对SCLC细胞生物学行为的影响，并为SCLC预后分子标志物提供新的靶点。

## 1 材料和方法

### 1.1 研究样本采集

收集2017年12月-2018年12月期间本院51例SCLC手术患者肿瘤标本及癌旁正常组织标本作为研究对象，其中男38例，女13例，平均（57.1±9.4）岁。见表 1。所有患者术前均未进行化学治疗，所有新鲜标本离体后，均迅速液氮冷冻保存，并经术后病理确诊。以上标本获得本院伦理委员会同意及家属签字认可，操作符合临床实验伦理规范。

**1:** 小细胞肺癌患者临床资料（*n*=51） Clinical data of patients with small cell lung cancer (*n*=51)

Characteristic	*n*	%
Gender		
Male	38	74.51
Female	13	25.49
Operative type		
Pulmonary lobectomy	25	49.02
Total pneumonectomy	11	21.57
Sleeve resection	9	17.65
Wedge resection	6	11.76
Pathological stage		
IIa	16	31.37
IIb	5	9.80
IIIa	20	39.22
IIIb	10	19.61
T stage		
T1	6	11.76
T2	29	56.86
T3	8	15.69
T4	8	15.69
N stage		
N0	6	11.76
N1	12	23.53
N2	33	64.71

### 1.2 细胞培养与转染

SCLC细胞株NCI-H1688和NCI-H446细胞用含10%胎牛血清、100 U/mL链霉素和100 U/mL青霉素的RPMI-1640培养基，置于37 ℃、5% CO_2_培养箱中培养。将生长状态良好的细胞铺于6孔板，约5×10^5^个/孔，待细胞在6孔板中的生长融合度至70%时，按照转染试剂Lipofectamine^TM^ 2000试剂说明书转染NC-inhibitor、miR-665 inhibitor或siLLGL1。

### 1.3 qRT-PCR

使用Trizol从细胞系和组织样品提取总RNA，按照反转录试剂盒的说明书将提取的总RNA反转成cDNA。按照qRT-PCR试剂盒（11791200, Thermo Fisher Scientific, Inc）说明书进行进行PCR反应。miR-665表达水平以U6为内参，LLGL1表达水平以GAPDH为内参，qRT-PCR结果以2^-△△CT^值计算得到。

### 1.4 双荧光素酶报告基因实验

采用TargetScan（http://www.targetscan.org）预测miR-665与LLGL1的潜在结合位点。构建LLGL1野生型3’-UTR荧光素酶报告基因质粒pMIR-LLGL1-wt和突变型报告基因质粒pMIR-LLGL1-Mut。在NCI-H1688细胞中共转染miR-NC或miR-665 mimics以及pMIR-LLGL1-wt或pMIR-LLGL1-Mut，在NCI-H446细胞中共转染NC-inhibitor或miR-665 inhibitor以及pMIR-LLGL1-wt或pMIR-LLGL1-Mut。转染24 h后，加入裂解液裂解15 min。使用双荧光素酶报告系统（Promega Corporation）分析了荧光素酶的活性。

### 1.5 Western blot

采用RIPA裂解液提取细胞或组织蛋白，遵照BCA法测定蛋白浓度，加入缓冲液后变性蛋白。按每泳道以50 μg蛋白进行12% SDS-PAGE电泳分离蛋白，将蛋白转移到PVDF膜上，5%脱脂奶粉37 ℃封闭1 h。加入一抗LLGL1抗体（1:500, ab39292, Abcam）和GAPDH抗体（1:1, 000, ab181602, Abcam），4 ℃孵育过夜。加入辣根过氧化物酶标记的二抗（1:1, 000, ab150077, Abcam），室温孵育1 h。ECL液暗室发光显影，采集图像并分析。

### 1.6 免疫组化

组织标本石蜡包埋后进行连续石蜡切片。石蜡切片置于65 ℃烤箱中过夜，然后梯度脱蜡、水化。在组织切片上加入3% H_2_O_2_，阻断内源性过氧化物酶。切片置于柠檬酸盐缓冲液并采用微波加热法以修复抗原。加入5%的正常羊血清封闭，室温孵育20 min。加入一抗LLGL1抗体（1:100），4 ℃过夜。加入辣根过氧化物酶标记的二抗（1:200），37 ℃孵育30 min。滴加辣根酶标记链霉卵白素工作液，37 ℃孵育30 min。采用DAB显色后，苏木精室温染色2 min，然后脱水和中性树脂封片。

### 1.7 CCK8法

NCI-H446细胞、NCI-H1688细胞按1×10^4^个/孔接种于96孔板，每孔200 µL细胞悬液，常规培养24 h后，参照CCK-8试剂盒操作说明书操作向每孔加入10 µL CCK-8溶液，37 ℃孵育1 h-2 h后，用酶标仪测定450 nm处的吸光度。

### 1.8 流式细胞法

NCI-H446细胞、NCI-H1688细胞用预冷的PBS重悬，将细胞悬液加入预冷的70%乙醇中固定，置于4 ℃过夜。去除乙醇后，加入100 μL RNase A，37 ℃水浴30 min，接着添加400 μL PI染色并混匀，4 ℃避光30 min，最后用流式细胞仪检测细胞周期。

### 1.9 Transwell迁移实验

NCI-H446细胞、NCI-H1688细胞用无血清悬浮细胞，调整细胞密度为1×10^6^个/mL，上室中加入100 μL细胞悬液；在下室加入600 μL含10%胎牛血清的培养基；置于37 ℃、5% CO_2_培养箱中培养24 h。取出小室后用PBS淋洗3次；将小室置于95%乙醇中固定5 min；在0.5%结晶紫染色液中染色10 min后，用PBS漂洗去除未结合细胞的染色液。用棉签轻拭去小室滤膜上层的细胞，在显微镜下观察滤膜下层细胞。侵袭实验：将Matrigel凝胶置于4 ℃过夜；次日将液化的Matrigel凝胶用无血清的细胞培养基以1:6的比例稀释。随后加入50 μL Matrigel稀释液到上室以包被滤膜；置于孵箱中4 h令包被液晾干。剩余步骤同Transwell细胞迁移实验。

### 1.10 细胞划痕实验

NCI-H446细胞、NCI-H1688细胞接种于6孔板上，当细胞汇合度达到80%-90%时，用200 μL的移液器枪头在正中央位置划一直线，形成单层细胞间的划痕。利用PBS冲洗将脱落的细胞冲洗掉，直至空中无脱落细胞存在。最后加入无血清的DMEM培养基，并分别于0 h、12 h显微镜下拍照，整理统计数据。

### 1.11 建立肺癌裸鼠移植瘤模型

选用BALB/c裸鼠12只，4周龄左右，体重13 g-17 g。动物饲养于22 ℃恒温，40%-75%湿度，12 h光/暗周期条件下，自由获取水和食物。所有裸鼠随机分为NC-inhibitor组（*n*=6）和miR-665 inhibitor组（*n*=6）。NCI-H446细胞转染NC-inhibitor或miR-665 inhibitor（B03001，上海吉玛制药技术有限公司）后，配置成浓度约为1×10^7^/mL单细胞悬液，并分别以0.2 mL/只接种于裸鼠左前上肢腋下处皮下。接种后定期观察，3周后采用颈椎脱臼法处死全部裸鼠，剥离瘤体，称重，拍照，甲醛固定。所有操作均获得河北医科大学第四医院实验动物福利伦理委员会同意，动物伦理编号为IACUC-4^th^ Hos Hebmu，操作符合动物实验伦理规范。

### 1.12 统计学方法

采用SPSS Statistics 22.0统计软件进行统计学分析，数据以均数±标准差（Mean±SD）表示，两组间以两单独样本*t*检验，三组或三组以上的比较采用*one-way ANOVA*，*P* < 0.01为差异显著。

## 2 结果

### 2.1 miR-665在肺癌组织中的表达以及对LLGL1的靶向调控

采用qRT-PCR检测肺癌组织和癌旁正常组织中miR-665的表达水平，如图 1A所示，miR-665在肺癌组织中的表达水平明显高于癌旁正常组织（*P* < 0.01）。采用TargetScan预测miR-665的潜在靶基因，结果显示，miR-665在LLGL1的3’-UTR区域具有潜在的结合位点（图 1B）。在NCI-H1688细胞中共转染pMIR-LLGL1-wt或pMIR-LLGL1-Mut以及miR-NC或miR-665 mimics后进行双荧光素酶报告基因实验，结果显示，共转染pMIR-LLGL1-wt和miR-665 mimics时，荧光素酶活性明显降低（*P* < 0.01），而共转染pMIR-LLGL1-Mut和miR-665 mimics时，荧光素酶活性无明显变化（图 1C）。将pMIR-LLGL1-wt或pMIR-LLGL1-Mut以及NC-inhibitor或miR-665 inhibitor共转染进NCI-H446细胞后发现，miR-665 inhibitor能明显增强pMIR-LLGL1-wt的荧光素酶活性（*P* < 0.01），而对pMIR-LLGL1-Mut的荧光素酶活性无明显影响（图 1D）。采用qRT-PCR和Western blot检测转染后的各组细胞中miR-665和LLGL1的表达水平，结果显示，转染miR-665mimics的NCI-H1688细胞中miR-665的表达水平较miR-NC对照组明显升高（*P* < 0.01），LLGL1的mRNA和蛋白表达明显降低（*P* < 0.01）。而转染miR-665 inhibitor的NCI-H446细胞中miR-665的表达水平较NC-inhibitor对照组明显降低（*P* < 0.01），LLGL1的mRNA和蛋白表达明显升高（*P* < 0.01）（图 1E-图 1G）。

**1:**
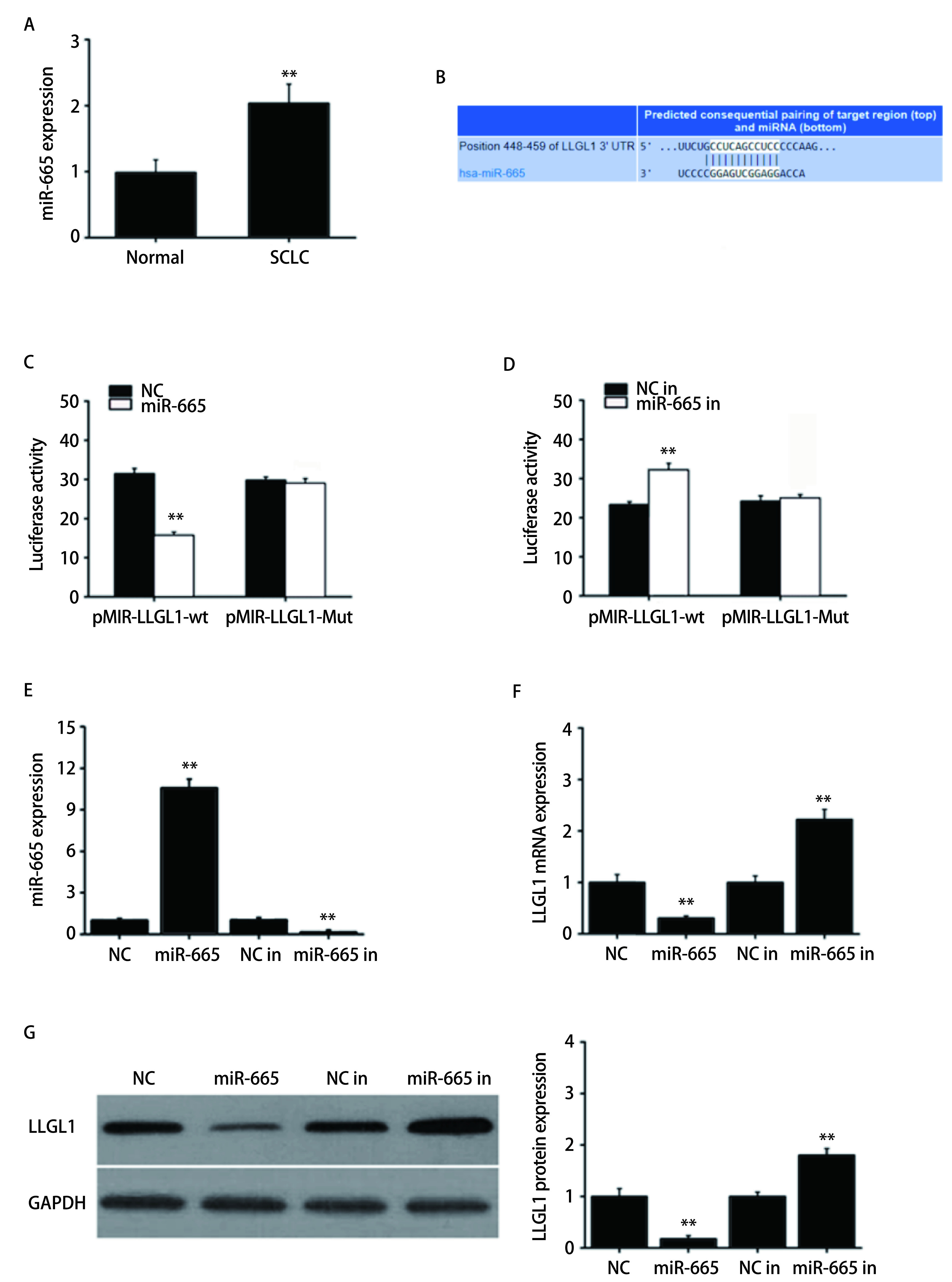
miR-665在肺癌组织中的表达以及对LLGL1的靶向调控。A：qRT-PCR检测肺癌组织和癌旁正常组织中miR-665的表达水平；B：TargetScan预测miR-665与LLGL1的潜在结合位点；C和D：双荧光素酶报告基因实验检测荧光素酶活性；E：qRT-PCR检测miR-665的表达水平；F：qRT-PCR检测LLGL1的mRNA表达水平。G：Western blot检测LLGL1蛋白的表达水平。**P < 0.01。 Expression of miR-665 in SCLC tissues and its targeting regulation of LLGL1. A: The expression of miR-665 in SCLC tissues and adjacent normal tissues was detected by qRT-PCR; B: TargetScan predicted potential binding sites for miR-665 and LLGL1; C and D: Double luciferase reporter assay was used to detect luciferase activity; E: The expression of miR-665 was detected by qRT-PCR; F: The mRNA expression of LLGL1 was detected by qRT-PCR; G: The expression of LLGL1 protein was detected by Western blot. **P < 0.01. SCLC: small cell lung cancer.

### 2.2 LLGL1在肺癌组织中的表达

采用免疫组化检测肺癌组织和癌旁正常组织中LLGL1的表达水平，如图 2A所示，相比癌旁正常组织，肺癌组织中LLGL1的阳性信号明显降低（*P* < 0.01）。qRT-PCR和Western blot检测结果也显示，肺癌组织中LLGL1的mRNA和蛋白表达明显低于癌旁正常组织（*P* < 0.01）（图 2B，图 2C）。

**2:**
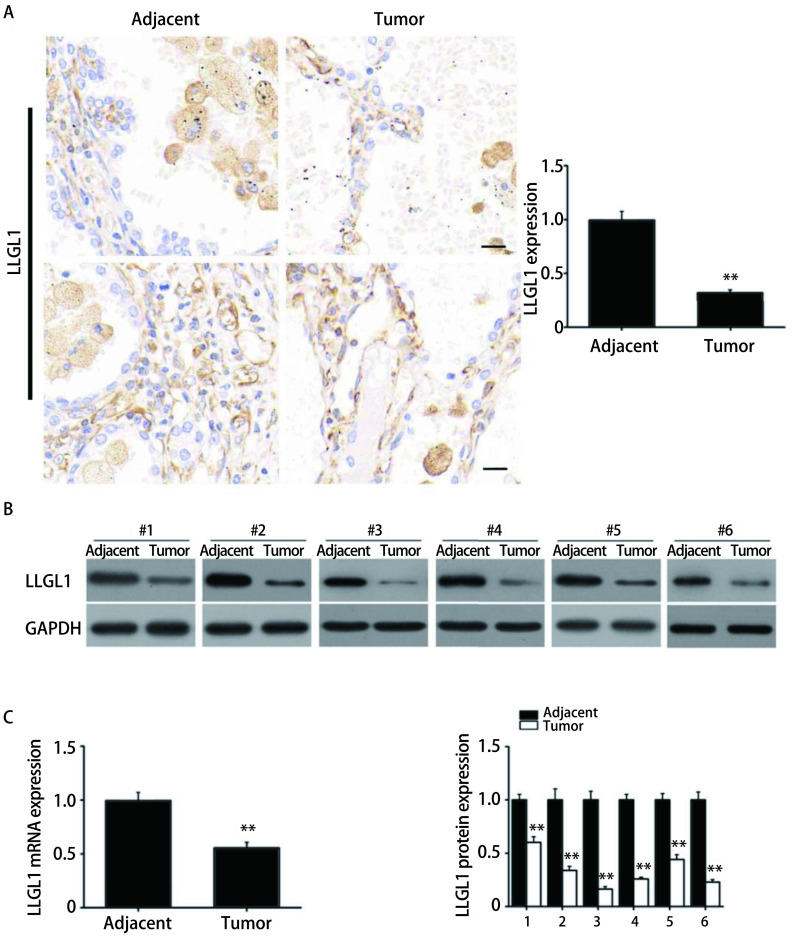
LLGL1在肺癌组织中的表达。A：免疫组化检测肺癌组织和癌旁正常组织中LLGL1的表达水平（bar=20 μm）；B：Western blot检测肺癌组织和癌旁正常组织中LLGL1的表达水平；C：qRT-PCR检测肺癌组织和癌旁正常组织中LLGL1的mRNA表达水平。**P < 0.01。 Expression of LLGL1 in SCLC tissue. A: The expression of LLGL1 in SCLC and normal tissues was detected by immunohistochemistry (bar=20 μm); B: The expression of LLGL1 in SCLC and normal tissues was detected by Western blot; C: The mRNA expression of LLGL1 in SCLC and normal tissues was detected by qRT-PCR. **P < 0.01.

### 2.3 miR-665和LLGL1对肺癌细胞增殖、周期、侵袭和迁移的影响

在NCI-H446细胞中转染NC-inhibitor或miR-665 inhibitor以及共转染miR-665 inhibitor和siLLGL1。采用CCK8法检测细胞转染后的增殖能力。如图 3A所示，转染miR-665 inhibitor的细胞增殖能力较对照组明显降低，而干扰LLGL1的表达后，增殖能力则明显升高（*P* < 0.01）。流式细胞法检测结果显示，抑制miR-665的表达能明显降低S期细胞比值，但干扰LLGL1的表达则能诱导S期阻滞（*P* < 0.01）（图 3B）。Transwell实验结果显示，转染miR-665 inhibitor能抑制细胞的侵袭及迁移能力，但siLLGL1能逆转这种抑制效果（*P* < 0.01）（图 3C）。采用细胞划痕实验对细胞的迁移能力检测结果同Transwell迁移实验结果一致（图 3D）。另一方面，在NCI-H1688细胞中转染miR-NC或miR-665 mimics以及共转染miR-665 mimics和LLGL1并重复上述实验，结果显示，过表达miR-665能明显促进细胞的的生物学行为，但这种促进效果会被LLGL1逆转（图 4A-图 4D）。

**3:**
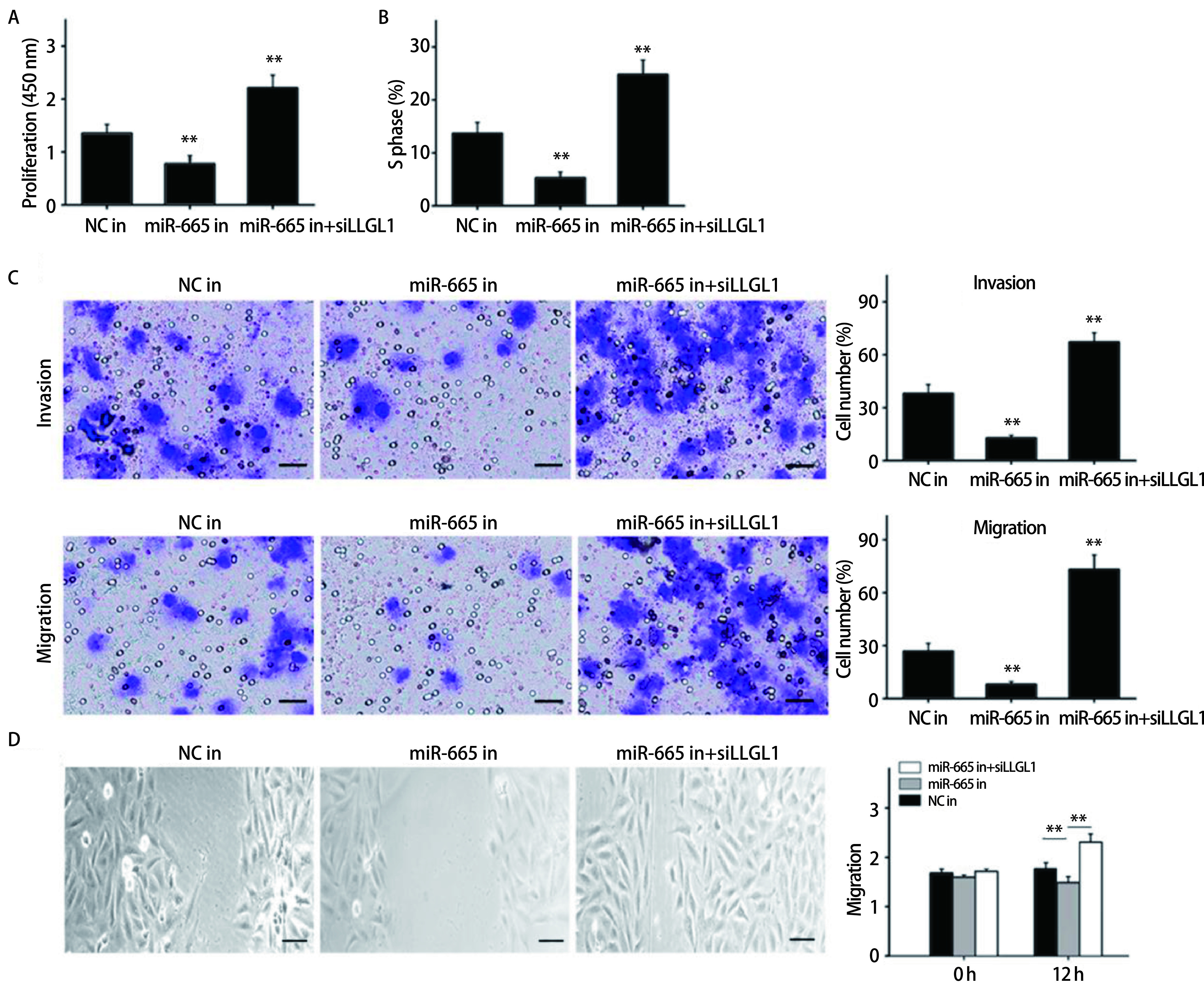
miR-665 inhibitor和siLLGL1对肺癌细胞增殖、周期、侵袭和迁移的影响。A：CCK8法检测细胞的增殖能力；B：流式细胞法检测细胞的S期比值；C：Transwell检测细胞的侵袭和迁移能力（bar=25 μm）；D：细胞划痕实验检测细胞的迁移能力（bar=25 μm）。**P < 0.01。 Effects of miR-665 inhibitor and siLLGL1 on proliferation, cycle, invasion and migration of SCLC cells. A: The proliferation ability of cells was detect by CCK8 assay; B: S-phase fraction of cells was detected by flow cytometry; C: The cell invasion and migration was detected by Transwell (bar=25 μm); D: The migration ability of cells was measured by wound healing (bar=25 μm). **P < 0.01.

**4:**
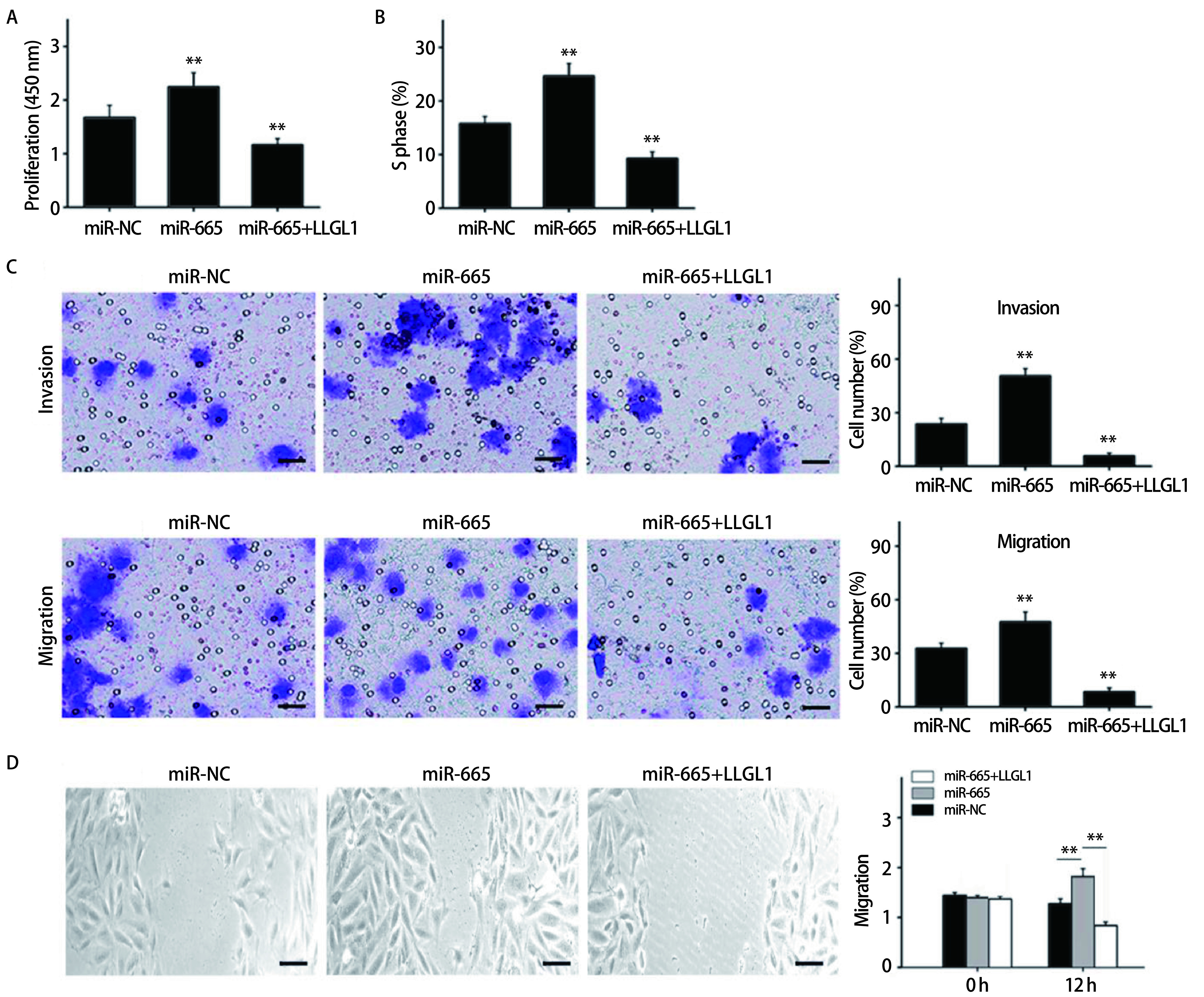
miR-665 mimics和LLGL1对肺癌细胞增殖、周期、侵袭和迁移的影响。A：CCK8法检测细胞的增殖能力；B：流式细胞法检测细胞的S期比值；C：Transwell检测细胞的侵袭和迁移能力（bar=25 μm）；D：细胞划痕实验检测细胞的迁移能力（bar=25 μm）。**P < 0.01。 Effects of miR-665 mimics and LLGL1 on proliferation, cycle, invasion and migration of SCLC cells. A: The proliferation ability of cells was detect by CCK8 assay; B: S-phase fraction of cells was detected by flow cytometry; C: The cell invasion and migration was detected by Transwell (bar=25 μm); D: The migration ability of cells was measured by wound healing (bar=25 μm). **P < 0.01.

### 2.4 miR-665在肺癌裸鼠移植瘤模型中的作用

将转染了NC-inhibitor或miR-665 inhibitor以及miR-NC或miR-665 mimics的NCI-H446细胞接种于裸鼠皮下构建肺癌裸鼠移植瘤模型。21 d后检测结果显示，相比对照组，miR-665 inhibitor组裸鼠的肿瘤体积和重量均明显降低（*P* < 0.01）（图 5A，图 5B，图 5G）。Western blot检测结果显示，miR-665 inhibitor组裸鼠肿瘤组织中LLGL1蛋白的表达明显降低于对照组（*P* < 0.01）（图 5C）。而过表达miR-665则产生相反的结果（图 5D-图 5F，图 5H）。

**5:**
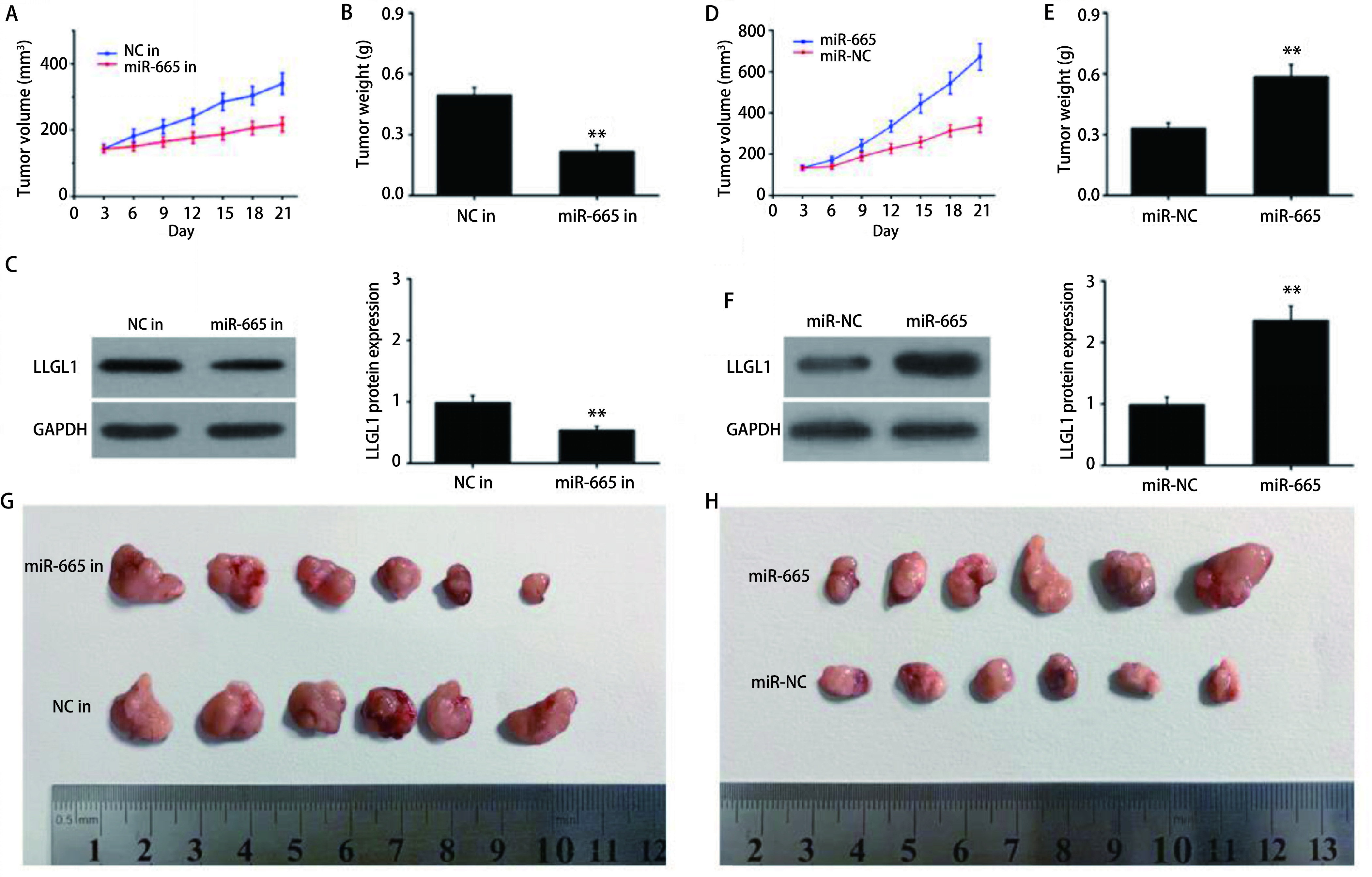
miR-665在肺癌裸鼠移植瘤模型中的作用。A和D：检测肿瘤体积；B和E：检测肿瘤重量；C和F：Western blot检测肿瘤组织中LLGL1蛋白的表达；G和H：各组移植瘤图片。**P < 0.01。 The role of miR-665 in a nude mouse xenograft model of SCLC. A and D: tumor volumes; B and E: tumor weights; C and F: the expression of LLGL1 protein in tumor tissues was detected by Western blot; G and H: the images of xenografts. **P < 0.01.

## 3 讨论

miRNA具有广泛的基因调节功能，在多种生物学活动包括细胞分化、增殖、凋亡和细胞周期改变等过程中发挥重要的调控作用，如果此过程中miRNA表达异常，将引起细胞生命进程的紊乱和疾病的发生^[11]^。近年来miR-655已证实与多种疾病的发生发展有关，Yang等^[12]^研究证明包括miR-655在内的7种miRNAs在2型糖尿病出现异常表达。Chang等^[13]^研究发现在食管鳞癌中miR-655呈现高表达，可以抑制食管鳞癌细胞增殖和侵袭，同时在患者的预后过程中发挥负调控作用。虽然目前已报道了多种miRNA同肺癌的发病机理有关^[14-16]^，但miR-665在SCLC中的作用尚鲜有报道。在本研究中，我们发现miR-665在SCLC组织中的表达水平明显高于癌旁正常组织，提示miR-665表达水平的异常变化可能和肺癌存在相关性。

miRNA通过与靶mRNA的3'-UTR的完全或不完全碱基配对结合而使靶mRNA降解或翻译抑制，对细胞分化、增殖、凋亡等生物过程进行调节，目前估计人类30%的基因可能被miRNA调控^[17, 18]^。因此，研究miRNA的作用机制，需首先要认识miRNA与其靶基因的相互作用。本研究通过TargetScan发现LLGL1的3’-UTR区域可能存在位点与miR-665形成互补结合。我们进一步通过双荧光素酶报告基因实验验证LLGL1是否为miR-665的靶基因，结果显示，miR-665 mimics能抑制pMIR-LLGL1-wt的荧光素酶活性，miR-665 inhibitor能增强pMIR-LLGL1-wt的荧光素酶活性，而两者对pMIR-LLGL1-Mut的荧光素酶活性均无明显影响。并且qRT-PCR和Western blot检测结果也证明了miR-665能负性调控LLGL1的mRNA和蛋白表达。这些结果表明miR-665能直接靶向作用于LLGL1。

Lgl最先在果蝇中被发现，其主要作用在于调节细胞极性，参与细胞分化、增殖、迁移、黏附和转化。LLGL1为Lgl的人类同源基因，有研究^[19]^显示，它的失活很可能与人类肿瘤的发生发展有密切关系。越来越多的证据表明LLGL1在各种人类肿瘤中起着抑制作用。Kuphal等^[20]^研究发现在黑色素瘤中LLGL1通过下调MMP2和MMP14的表达并促进E-钙粘蛋白的表达来促进细胞黏附、抑制细胞迁移。在本研究中，qRT-PCR、Western blot和免疫组化实验结果均显示LLGL1在肺癌组织中的表达水平较癌旁正常组织明显降低，这与以往研究结果类似，提示其具有肿瘤抑制作用。为分析miR-665对肺癌细胞生物学行为的影响及其作用机制，我们利用NCI-H446细胞、NCI-H1688细胞进行了一系列细胞实验。结果显示，抑制miR-665的表达可以抑制肺癌NCI-H446细胞的增殖、S期细胞比值、侵袭和迁移能力，但是干扰LLGL1能逆转这种抑制效果，随后我们上调miR-665的表达，发现可以促进肺癌NCI-H1688的增殖、S期细胞比值、侵袭以及迁移能力，但这种促进效果同样被LLGL1的过表达逆转。这提示我们miR-665是通过靶向调控LLGL1从而促进肺癌细胞生物学行为。为进一步验证miR-665在SCLC中的作用，本研究构建了肺癌裸鼠移植瘤模型。我们观察到抑制miR-665能上调LLGL1蛋白的表达并抑制肿瘤的生长。但过表达miR-665则产生相反的效果。该结果与体外实验一致，表明miR-665在肺癌中发挥促癌基因的作用。

综上所述，本研究观察到miR-665在SCLC组织中存在异常高表达，下调其表达能抑制肺癌细胞的增殖、S期阻滞、侵袭和迁移，抑制肺癌裸鼠移植瘤的生长。并进一步阐明了miR-665是通过靶向调控LLGL1在SCLC中发挥促癌基因的作用。
